# HOW TO INTRODUCE A NEW TB VACCINE IN ADOLESCENTS AND ADULTS: Insights from Key Stakeholders in Mozambique, Southern Africa

**DOI:** 10.64898/2026.05.10.26352803

**Published:** 2026-05-13

**Authors:** Agostinho Viana Lima, Doyoon Kim, Sozinho Acácio, Quinhas Fernandes, Benedita José, Benjamin Lopman, Alberto L. García-Basteiro, Kristin N. Nelson

## Abstract

Tuberculosis (TB) remains a major global health challenge, particularly in low- and middle-income countries such as Mozambique. To address this burden, promising new preventive TB vaccines targeting adolescents and adults are currently in phase III efficacy trials. This study aimed to assess stakeholders’ perspectives on priority high-risk groups, the challenges in reaching them, and potential strategies for delivering a TB vaccine. We conducted a qualitative study using semi-structured interviews with members of the National TB Program, the National Immunization Program, and the National Immunization Technical Advisory Group. Data were collected between March and July 2024. Our findings suggest that a TB vaccine program in Mozambique should prioritize individuals with comorbidities, especially those living with HIV or diabetes, and close contacts of TB patients, followed by healthcare workers, miners, and incarcerated populations. Although uptake is expected to vary across groups, relatively high coverage was anticipated among people living with HIV, TB contacts, and older adults, as well as healthcare workers, incarcerated individuals, formal miners, and in-school adolescents. To improve uptake, campaign-based strategies using mobile brigades were considered promising approaches to expand coverage. Stakeholder perspectives highlight the importance of prioritizing high-risk groups and adopting context-specific delivery strategies to support the effective introduction of a TB vaccine in Mozambique.

**Clinical trial number:**

not applicable.

## Full Text Availability

The license terms selected by the author(s) for this preprint version do not permit archiving in PMC. The full text is available from the preprint server.

